# Differential Cerebrovascular Toxicity of Various Tobacco Products: A Regulatory Perspective

**DOI:** 10.4172/2329-6887.1000e130

**Published:** 2015-01-09

**Authors:** Ravi K Sajja, Pooja Naik, Luca Cucullo

**Affiliations:** Department of Pharmaceutical Sciences, Texas Tech University Health Sciences Center, Amarillo, USA

## Editorial

Blood-Brain Barrier (BBB) is a dynamic anatomical interface that separates brain parenchyma from blood circulation and is principally constituted by the cerebral microcapillary endothelial cells [1,2]. BBB is composed of distinct structural and functional organization through the presence of inter-endothelial tight junction complexes, abundant expression of nutrient and efflux transporters including metabolically active sites. While the TJ complexes tightly seal the paracellular gaps and contribute to high resistance of BBB [3], the presence of specific nutrient transporters and receptor systems selectively regulate the delivery of metabolic substrates, nutrients and macromolecules to the brain. In addition, efflux transporters belonging to the ABC superfamily prevent the brain permeation of blood-borne neurotoxic chemicals including xenobiotics and eliminates the accumulation of toxic metabolites within the brain parenchyma [1]. Taken together, the BBB serves as a physiological, transport and metabolic barrier that critically regulates ion, molecular and cellular flux into the brain, thus maintaining the CNS microenvironment for optimal neuronal function [2]. Importantly, impairment of BBB integrity by various exogenous or endogenous pathological stimuli involving increased load of oxidative/inflammatory stress in the neurovascular unit, is a potential mechanism underlying the pathogenesis of a host of neurologic and degenerative disorders [3,4].

Tobacco smoking is a serious public health concern and a leading cause of increased rates of preventable mortality and morbidity across the globe with a significant toll on health care costs and personal life (1% of deaths world-wide annually). Cigarette smoke contains a complex mixture of > 8000 chemicals in vapor and particulate phases such as reactive aldehydes and pro-oxidants with high potential for cellular oxidative injury. Tobacco Smoke (TS) is a potential risk factor in the pathogenesis of various systemic disorders such as cardiovascular diseases, atherosclerosis, insulin resistance and other vascular abnormalities [5]. Mounting evidence suggests that mainstream TS elicits a strong inflammatory and immune response [6], leading to potential endothelial dysfunction and microvascular damage [5]. In addition, both active and passive smoking aggravates endothelial cell activation with significant increase in endothelial-leukocyte interactions leading to profound monocyte infiltration and local inflammation [5,7].

Emerging evidence also indicts TS (active and passive) as preventable risk factor in the etiology of neurological disorders such as ischemic stroke, silent cerebral infarctions, multiple sclerosis and cerebral aneurysms [8–10]. Although the precise mechanisms implicated in TS induced neuropathogenesis is not completely understood, nicotine-dependent pathways at the cerebrovasculature are believed to partly mediate TS-induced neurotoxicity and stroke [11]. Importantly, main stream TS was shown to impair BBB dysfunction that was prevented by anti-oxidant supplementation [7], while nicotine exacerbated ischemic stroke and brain edema [12]. Further, nicotine was also shown to alter BBB permeability *in vivo* [13] and aggravated post-ischemic inflammatory responses [14]. Thus, it is proposed that BBB damage is a critical cerebrovascular complication of TS-induced oxidative/inflammatory stress, prodromal to pathogenesis of a host of CNS disorders [7,9].

Currently, various tobacco products are being commercially marketed such as ‘reduced exposure’, ‘light’ or ‘nicotine-free’ products including electronic cigarettes with different levels of nicotine, nitrosamines and other toxic chemicals, claiming a reduced health hazard compared to regular brands. However, experimental and clinical evidence assessing the toxicological profiles of these diverse tobacco products at the brain microvasculature is minimally reported to support their safety and availability of such information is deemed to be essential for regulatory bodies to set standards on the tobacco products for improving public health [15]. Recent studies from our laboratory and others have challenged the safety of these reduced or low-exposure products. For example, recently we investigated the toxic impact of mainstream TS (whole) extracts from various tobacco products on BBB endothelium in vitro [16]. Our data revealed a strong positive correlation between the TS-induced BBB endothelial dysfunction (through increased oxidative stress) and total tar and nitric oxide content of various tobacco products (such as regular full flavor 3R4F, Ultra low nicotine, Ultra-Light 1R5F, and Nicotine-Free). Mainly, smoke extracts from Nicotine-Free and Ultra low nicotine cigarettes were found to be more toxic at BBB endothelium compared to regular products [16].

In summary, rigorous analysis of the safety (or toxicity) profiles of various tobacco products on cerebrovascular function in experimental and clinical studies is critically required for regulatory control of these diverse tobacco products. In addition, such studies would provide mechanistic insights into the molecular mechanisms underlying tobacco smoke associated BB neurotoxicity.
